# Identification of α-N-catenin as a novel tumor suppressor in neuroblastoma

**DOI:** 10.18632/oncotarget.27096

**Published:** 2019-08-20

**Authors:** Jingbo Qiao, Eric J. Rellinger, Kwang Woon Kim, Camille M. Powers, Sora Lee, Hernan Correa, Dai H. Chung

**Affiliations:** ^1^ Department of Surgery, UT Southwestern Medical Center, Dallas, TX 75390, USA; ^2^ Department of Pediatric Surgery, Vanderbilt University Medical Center, Nashville, TN 37232, USA; ^3^ Department of Pathology, Vanderbilt University Medical Center, Nashville, TN 37232, USA

**Keywords:** neuroblastoma, tumor suppressor, CTNNA2 (α-N-catenin), NF-κB

## Abstract

The lost expression of α-catenin has been found in cancers, and reinstalling α-catenin inhibits tumor growth. Here we hypothesized that the α-N-catenin, a homologous member of α-catenin and neural-specific expressed, functions as a novel tumor suppressor in neural crest-derived tumor, neuroblastoma. We correlated CTNNA2 (encodes α-N-catenin) expression to neuroblastoma disease relapse-free survival probability using publicly accessible human neuroblastoma datasets in R2 platform. The result showed that it negatively correlated to relapse-free survival probability significantly in patients with neuroblastoma with non-MYCN amplified tumor. Conversely, overexpressing CTNNA2 suppressed the neuroblastoma cell proliferation as measuring by the clonogenesis, inhibited anchorage-independent growth with soft agar colony formation assay. Forced expression of CTNNA2 decreased cell migration and invasion. Further, overexpression of CTNNA2 reduced the secretion of angiogenic factor IL-8 and HUVEC tubule formation. Our results show, for the first time, that α-N-catenin is a tumor suppressor in neuroblastoma cells. These findings were further corroborated with *in vivo* tumor xenograft study, in which α-N-catenin inhibited tumor growth and reduced tumor blood vessel formation. Interestingly, this is only observed in SK-N-AS xenografts lacking MYCN expression, and not in BE(2)-C xenografts with MYCN amplification. Mechanistically, α-N-catenin attenuated NF-κB responsive genes by inhibiting NF-κB transcriptional activity. In conclusion, these data demonstrate that α-N-catenin is a tumor suppressor in non-MYCN-amplified neuroblastomas and it inhibits NF-κB signaling pathway to suppress tumor growth in human neuroblastomas. Therefore, restoring the expression of α-N-catenin can be a novel therapeutic approach for neuroblastoma patients who have the deletion of CTNNA2 and lack of MYCN amplification.

## INTRODUCTION

Neuroblastoma is a highly virulent extracranial neural crest-derived solid tumor that affects infants and children. The development of multi-modality therapy has made improvements in the overall survival of patients with neuroblastoma; however, those high-risk group of patients presenting with advanced-stage tumors at diagnosis remain difficult to cure with a dismal long-term overall survival of less than 50%.

Genetic alterations have been identified in neuroblastomas, including segmental chromosome 1p, 3p, 4p, and 11q deletions, and 1q and 17q gain; gene mutations including MYCN amplification, ALK amplification and mutation, and LIN28B amplification and polymorphism. These genetic aberrations are associated with disease initiation and progression in neuroblastoma, and correlated with a clinical outcome such as overall mortality rate [1]. Alterations in epigenetic regulation recently also have been identified in neuroblastoma [2].

Here, we identified one novel tumor suppressor, α-N-catenin (CTNNA2), a cell adhesion molecule, silenced in some non-MYCN amplified neuroblastomas, correlated to low event-free survival probability. Alpha-catenin is a cell adhesion protein and plays critical roles in intercellular adhesions including having a role in both stable and dynamic morphogenetic movements by binding with F-actin [3]. There are three members in a human α-catenin family, α-E-catenin (CTNNA1), α-N-catenin (CTNNA2), and α-T-catenin (CTNNA3). Among three isoforms, CTNNA2 expression has been shown to be neurally restricted [4] and α-N-catenin governs the stability of synaptic contact [5]. Meanwhile, dysregulation of constitutive α-catenin expressions has been found in various cancers: loss of α-catenin in prostate cancer [6], frameshift in breast cancer [7], and mutant in laryngeal carcinomas [8]. Likewise, the abnormal expression of intercellular adhesion protein can lead to a variety of pathologies, including tumorigenesis, and metastasis and hyperproliferation [9]. However, the exact role of CTNNA2 in neuroblastoma remains unknown.

Multiple signaling pathways are downstream of the cell adhesion molecules, such as β-catenin/Wnt signaling [6], Ras/MAPK signaling [10], the Hedgehog pathway [11], and the Hippo signaling pathway [12, 13]. Alpha-Catenin attenuated the effect of Src phosphorylation by increasing β-catenin association with E-cadherin and increased the sensitivity of prostate cancer cells to the Src inhibitor in suppressing cell proliferation [6]. Overexpression of full-length α-catenin attenuates Wnt signaling in *Xenopus* [14, 15]. Others reported that α-catenin suppressed tumor growth by inhibiting NF-κB signaling in E-cadherin-negative basal-like breast cancer cells [16]. However, neural-specific α-N-catenin has not been widely studied, especially in neural crest cell-derived tumor neuroblastomas. In this study, we investigated the role of α-N-catenin in neuroblastomas and identified that α-N-catenin acts as a tumor suppressor by inhibiting tumor aggressiveness and tumor angiogenesis.

## RESULTS

### α-N-catenin is a potential tumor suppressor in non-MYCN-amplified human neuroblastoma

**Figure 1:**
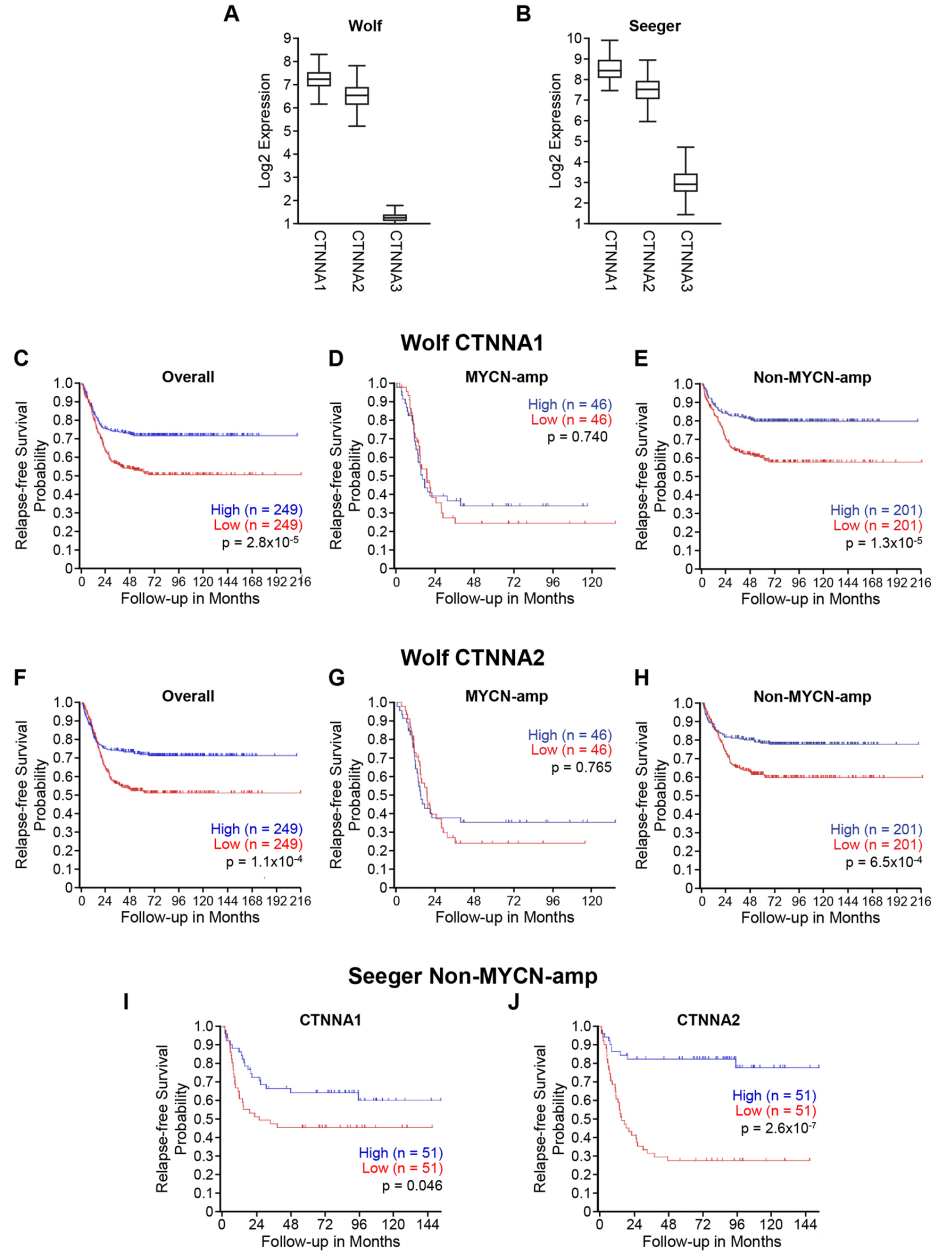
Low CTNNA2 expression is associated with disease relapse and mortality in patients with neuroblastoma lacking MYCN amplification. We analyzed the expressions of three CTNNA members in two datasets by utilizing the R2 analysis and visualization platform (http://r2.amc.nl). (**A**) The expression levels of CTNNA1 and CTNNA2 were higher than CTNNA3 in Wolf’s dataset which is a gene expression profiles generated from 498 primary neuroblastomas using RNA-Seq and microarrays. (**B**) The similar result showed in Seeger’s dataset which is a gene expression profiles of primary tumors from 102 patients with metastatic neuroblastoma lacking MYCN amplification. (**C**) CTNNA1 expression was analyzed in the overall of Wolf’s dataset using Kaplan scan. Kaplan–Meier curves showed the lower probability of relapse-free survival associated with lower expression of CTNNA1 (**D**) CTNNA1 expression was analyzed in MYCN-amplified subgroup of Wolf’s dataset using Kaplan scan. No significant relation between a probability of relapse-free survival and CTNNA1 expression (**E**) CTNNA1 expression was analyzed in non-MYCN-amplified subgroup of Wolf’s dataset using Kaplan scan. Kaplan–Meier curves showed the lower probability of relapse-free survival associated with lower expression of CTNNA1 (**F**) CTNNA2 expression was analyzed in the overall of Wolf’s dataset using Kaplan scan. Kaplan–Meier curves showed the lower probability of relapse-free survival associated with lower expression of CTNNA2 (**G**) CTNNA2 expression was analyzed in MYCN-amplified subgroup of Wolf’s dataset using Kaplan scan. No significant relation between a probability of relapse-free survival and CTNNA2 expression (**H**) CTNNA2 expression was analyzed in non MYCN-amplified subgroup of Wolf’s dataset using Kaplan scan. Kaplan–Meier curves showed the lower probability of relapse-free survival associated with lower expression of CTNNA2 (**I**) CTNNA1 expression was analyzed in Seeger’s dataset using Kaplan scan. Kaplan–Meier curves showed the lower probability of relapse-free survival associated with lower expression of CTNNA1 (**J**) CTNNA2 expression was analyzed in Seeger’s dataset using Kaplan scan. Kaplan–Meier curves showed the lower probability of relapse-free survival associated with lower expression of CTNNA2.

α-catenin has been known to play tumor suppressor roles in cancer [16] and lost, or decreased expression of α-catenin is often found in cancers [8]. However, much is unknown about α-N-catenin, which has a tissue-specific expression in neuronal tissues, especially in neuroblastoma. First, we utilized publicly accessible clinical datasets R2 Analysis and Visualization Platform (http://r2.amc.nl) and analyzed the expression of three CTNNA family members and their correlation to relapse-free survival probability in neuroblastomas. Two publicly available datasets: Wolf (generated gene expression profiles from 498 primary neuroblastomas using RNA-Seq and microarray) and Seeger (generated gene expression profiles of primary tumors from 102 patients with metastatic neuroblastoma lacking MYCN amplification) were used for this study. We found that CTNNA1 and CTNNA2 had higher expressions, while CTNNA3 had consistently lower expression in both Wolf and Seeger datasets from patients with neuroblastoma (Figure 1A and 1B).

Thus, we focused on CTNNA1 and CTNNA2 for further evaluation using the R2 platform. We analyzed the relationship between CTNNA1 and CTNNA2 expression and patient prognoses in large, independent cohorts featuring all stages of neuroblastoma. Specifically, we utilized Wolf’s neuroblastoma dataset, which has gene expression profiles generated from 498 primary neuroblastomas using RNA-Seq and microarrays to make a Kaplan-Meier Curve based upon CTNNA1 and CTNNA2 gene expression utilizing the R2: microarray analysis and visualization platform [17, 18]. The lower expressions of both CTNNA1 and CTNNA2 were associated with a decreased probability of relapse-free survival in overall patients as shown in Figure 1C and 1F, respectively. We further analyzed the correlation between the expressions of CTNNA1 and CTNNA2 and relapse-free survival in two subgroups based on the MYCN status: MYCN-amplified and non-MYCN-amplified groups. We found that expression of CTNNA1 and CTNNA2 correlated to relapse-free survival only in non-MYCN-amplified groups (Figure 1E and 1H), but not in MYCN-amplified groups (Figure 1D and 1G). Finally, we analyzed the expression of CTNNA1 and CTNNA2 in Seeger’s dataset, which contains 102 gene expression profiles from patients with metastatic neuroblastoma lacking MYCN-amplification. We also found that lower expression of CTNNA2 was associated with a decrease of relapse-free survival (Figure 1J), compared to CTNNA1 (Figure 1I, p=0.046). Therefore, we deduced that CTNNA2 is a potential tumor suppressor in neuroblastomas that lacked MYCN expression. Similar results were obtained by analyzing the datasets of Versteeg and Kocak (Supplementary Figure 1). Interestingly, if we analyze the data based on the clinical risk group, the results show that the expression of CTNNA2 at stage 4 is decreased significantly compared to other stages (Supplementary Figure 2A and 2B). Again, the sample size of MYCN-amplified group was limited for statistical analysis (Supplementary Figure 2C). Together, these data demonstrate that decreased levels of CTNNA2 are worse and associated with disease relapse and mortality in neuroblastoma patients, especially in those with neuroblastoma lacking MYCN-amplification.

### Forced expression of α-N-catenin suppressed the proliferation and anchorage-independent growth of neuroblastoma cells

**Figure 2:**
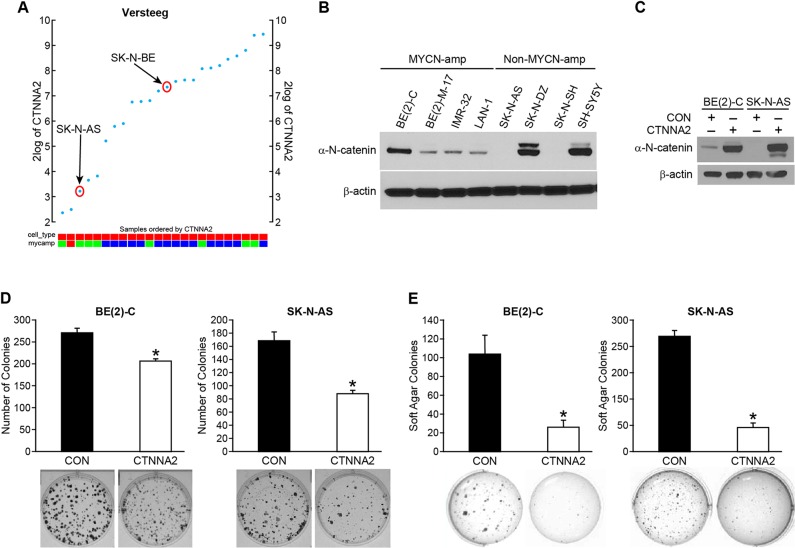
α-N-catenin inhibits colony formation and anchorage-independent growth of human neuroblastoma cells. (**A**) The expression of CTNNA2 was analyzed in 24 neuroblastoma cell lines from Versteeg’s dataset which contains gene expression profiles from neuroblastoma cell lines using the R2 platform. (**B**) α-N-catenin protein expression was detected in 8 human neuroblastoma cell lines. β-actin was used as an internal control. (**C**) BE(2)-C and SK-N-AS cells transfected with control vector (CON) and CTNNA2 overexpression vectors. Overexpression of α-N-catenin was confirmed in both BE(2)-C and SK-N-AS cells. β-actin was used as an internal control. (**D**) Clonogenesis assay was performed in BE(2)-C and SK-N-AS cells transfected with a control vector and CTNNA2 overexpression vectors. Bottom figures are representative images of colony formed from single cell proliferation. (**E**) Anchorage-independent assay was performed by quantifying the soft agar colony formed by BE(2)-C and SK-N-AS cells transfected with a control vector and CTNNA2 overexpression vectors. Data represent mean ± SEM; ^*^ = *p* < 0.05 vs. CON.

To investigate the role of α-N-catenin on cell aggressiveness, we performed functional assays. First, we analyzed the expression of CTNNA2 in Versteeg’s dataset which contains gene expression profiles from 24 neuroblastoma cell lines. As shown in Figure 2A, the expression level of CTNNA2 was found in varying levels in all cell lines tested. We examined the expression level of α-N-catenin in eight human neuroblastoma cell lines by immunoblotting, the expression levels are varied as shown in Figure 2B; the negative expression of α-N-catenin in SK-N-AS and SK-N-SH cell lines, moderate levels of α-N-catenin in BE(2)-M17, IMR-32, and LAN-1, and abundant expression of α-N-catenin in BE(2)-C, SK-N-DZ, and SH-SY5Y. There was no correlative expression shown between MYCN and CTNNA2 in neuroblastoma cell lines (Figure 2B). Based on the data in Figure 2A and 2B, we then chose two cell lines, which we used and successfully established animal xenograft models in our laboratory: SK-N-AS cells have no expression of CTNNA2 and are non-MYCN amplified while BE(2)-C cells have relatively higher expression of CTNNA2 and are MYCN amplified. We established stably-overexpressed CTNNA2 cells in both cell lines and confirmed the levels of CTNNA2 (Figure 2C). Next, we performed the clonogenic assay and anchorage-independent assay using both stable cell lines and our results showed that enhanced α-N-catenin significantly reduced cell proliferation to 77% and 52% in BE(2)-C and SK-N-AS cell lines respectively by clonogenesis assay (Figure 2D), indicating α-N-catenin negatively regulates tumor growth in neuroblastoma cells. Moreover, CTNNA2 significantly inhibited the anchorage-independent growth in the soft agar colony formation assay to 25% and 22% in BE(2)-C and SK-N-AS cells, respectively (Figure 2E). The ability of anchorage-independent growth correlates strongly with tumorigenicity and invasiveness. Thus, our data indicated that α-N-catenin played a tumor suppressor role in neuroblastoma cells.

### Forced expression of α-N-catenin decreased the migration, invasion, and angiogenesis *in vivo*

**Figure 3:**
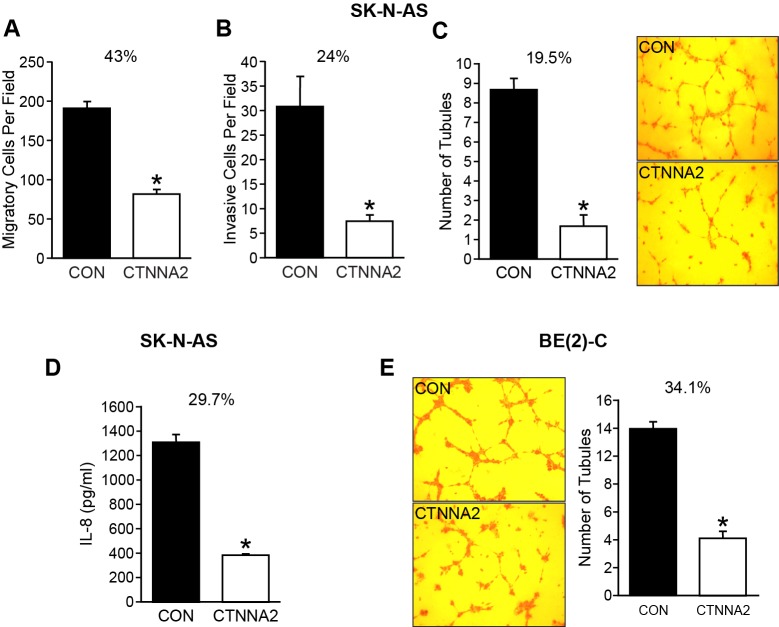
α-N-catenin inhibits migration, invasion, and angiogenesis *in vitro*. (**A**) SK-N-AS cells were plated in collagen type I-coated upper chamber of transwell (1x10^5^ cells/well) in serum-free media with the bottom well containing 10% FBS RIPM medium. After 6 h incubation, cells were fixed with 4% paraformaldehyde, stained with DAPI, and migrated cells were counted. Alpha-N-catenin overexpression resulted in a significant reduction in cell migration to 43% of CON cells (^*^ = *p* < 0.05 vs CON). (**B**) Cells (1.5×10^5^/well) in serum-free media were added to the upper well coated with Matrigel on the transwell filters and 10% FBS containing RPMI media added into the bottom well. After 48 h incubation, cells were fixed with 4% paraformaldehyde, stained with DAPI, and invaded cells were counted. Alpha-N-catenin overexpression resulted in a significant reduction in cell migration to 24% of CON cells (^*^ = *p* < 0.05 vs CON). (**C**) HUVECs were cultured in conditioned media collected from SK-N-AS cells transfected with a control vector and CTNNA2 overexpression vectors on Matrigel-coated 24-well plates for 6 h. Tubule staining was performed in triplicate and quantified. The number of tubules formed with HUVECs in conditioned media from α-N-catenin overexpressed cells was decreased to 19.5% of control. (Mean ± SEM; ^*^ = *p* < 0.005 vs. CON). (**D**) IL-8 secretion was measured in conditioned media from cells. Cells (3 × 10^5^ cells/ well) were plated in 6-well plate and cultured for 24 h, then the supernatants were collected for measuring IL-8 secretion by ELISA. α-N-catenin overexpression significantly inhibited IL-8 secretion down to 29.7% of control cells in conditioned media. Data represent mean ± SEM; ^*^ = *p* < 0.05 vs. CON. (**E**) HUVECs were cultured in conditioned media collected from BE(2)-C cells transfected with a control vector and CTNNA2 overexpression vectors on Matrigel-coated 24-well plates for 6 h. Tubule staining was performed in triplicate and quantified. The number of tubules formed with HUVECs in conditioned media from α-N-catenin overexpressed cells was decreased to 34.2% of control. (Mean ± SEM; ^*^ = *p* < 0.005 vs. CON).

Cell migration is a crucial step for a variety of morphogenetic events and pathogenetic processes such as cancer invasion [19]. α-catenin is localized in adherens junctions and is activated during cell migration by activating RhoA to increase actomyosin contractility at cell-cell junctions [20]. It has been reported that catenins steer cell migration via stabilization of front-rear polarity [21]. Therefore, loss of α-catenin can result in loss of cell-cell adhesion, a common characteristic of cancer cells. α-E-catenin (CTNNA1) has been well studied, however, neural-specific α-N-catenin has not been investigated yet. Thus, we examined the role of α-N-catenin on migration, invasion, and angiogenesis in neuroblastoma cells. Our results showed that both migration and invasion were decreased to 43% and 24%, respectively, by overexpression of α-N-catenin in SK-N-AS cells (Figure 3A and 3B), but no significant difference was observed in BE(2)-C cells, which further highlighted the MYCN independent tumor suppressor role of α-N-catenin in neuroblastoma cells.

We also evaluated the potential effect of α-N-catenin on tumor angiogenesis. We performed a tubule formation assay using Human umbilical vein endothelial cells (HUVECs), which is a rapid and quantitative *in vitro* method used for determining genes and pathways involved in angiogenesis. Our results indicated a significant difference in tubule formation. The tubule formed with conditioned media from α-N-catenin-overexpressed cells down to 19.5% of control cells without α-N-catenin expression (Figure 3C), suggesting CTNNA2 overexpressing cells decreased the secretion of angiogenic factors into their culture media in comparison to the control cells. We also collected the media from control cells and α-N-catenin overexpressed cells and measured secreted IL-8 using ELISA, and found that α-N-catenin inhibited the secretion of IL-8 down to 29.7% of control cells in SK-N-AS cell line (Figure 3D). Similarly, the tubule formation with HUVECs was decreased with conditional media from BE(2)-C cells with enhanced α-N-catenin expression down to 34.1% of control cells (Figure 3E). These results indicated that the level of α-N-catenin is inversely proportional to migration, invasion, and angiogenesis as well, further demonstrating the tumor suppressor role of α-N-catenin in neuroblastoma.

### α-N-catenin functions as a tumor suppressor and it inhibited tumor growth in animal xenografts

**Figure 4:**
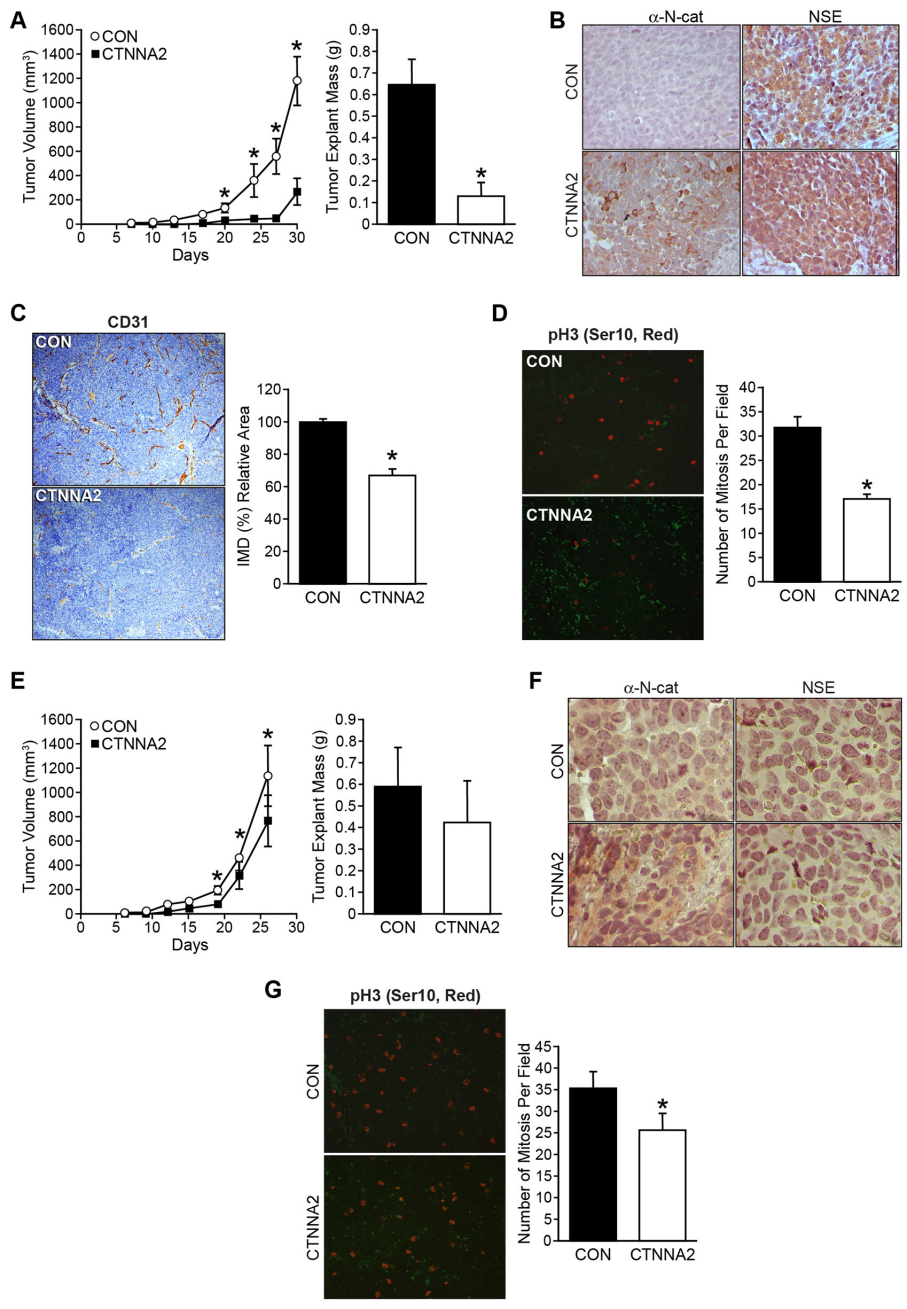
α-N-catenin inhibits tumor growth in tumor xenograft mouse model. (**A**) Mouse subcutaneous xenografts were established with SK-N-AS cells stably transfected with a control vector (CON) and CTNNA2 overexpression vectors in each flank side of the mouse (n = 10, Left). Tumor volume was measured biweekly. Tumor explant weights were obtained at the time of sacrifice at day 30 (Right). (**B**) Expression of α-N-catenin and NSE were detected by immunohistochemistry in tumor tissues from SK-N-AS xenografts. (**C**) Representative microphotographs of CD31 expression in SK-N-AS xenografts from control- or CTNNA2 overexpressed mice. CD31 was immunohistochemically stained with anti-human CD31 antibody (brown); magnification 100 ×. Intratumoral microvessel density was assessed by quantifying endothelial cells, using CD31-stained sections. (**D**) Mitotic cells were quantified by counting positive immunohistochemical stained phospho-Histone H3 (red) cells in paraffin-embedded sections from SK-N-AS xenografts. (**E**) Mouse subcutaneous xenografts were established with BE(2)-C cells stably transfected with a control vector (CON) and CTNNA2 overexpression vectors in each flank side of the mouse (n = 10, Left). Tumor volume was measured biweekly. Tumor explant weights were obtained at the time of sacrifice at day 26 (Right). (**F**) Expression of α-N-catenin and NSE were detected by immunohistochemical staining in tumor tissues from BE(2)-C xenografts. (**G**) Mitotic cells were quantified by counting positive immunohistochemical stained phospho-Histone H3 (red) cells in paraffin-embedded sections from BE(2)-C xenografts. Data represent mean ± SEM; ^*^ = *p* < 0.05 vs. CON.

We next sought to confirm whether CTNNA2 acts as a tumor suppressor in *in vivo*. To explore the function of α-N-catenin *in vivo*, we subcutaneously injected cells with CTNNA2 overexpression and their controls into athymic nude mice for tumor growth analysis. Strikingly, CTNNA2-overexpressed SK-N-AS cells had delayed tumor formation and smaller tumor volumes throughout the experiments than mice implanted with control cells (Figure 4A, tumor volume, left; tumor mass, right). The expressions of CTNNA2 and NSE, a neuronal marker, in tumor tissues were confirmed in dissected tissues using immunohistochemical staining (Figure 4B). To confirm CTNNA2 mediated angiogenesis inhibition *in vivo*, we performed CD31/PECAM-1 (Platelet Endothelial Cell Adhesion Molecule-1) immunohistochemical staining on tumor sections from mice and counted the blood vessels. The results demonstrated an approximately 33% reduction of the number of CD31-stained vessels in CTNNA2 tumors in comparison to tumors from control mice (Figure 4C). Also, we evaluated cell proliferation in tumors by staining tumor sections with the anti-human phospho-Histone H3 (ser10) antibody, followed by Alexa Flour 568 Dye. Phospho-Histone H3 (Ser10) is a cell mitosis marker that is tightly correlated with chromosome condensation during both mitosis and meiosis. As shown in Figure 4D, there were 50% less mitotic cells in the CTNNA2 tumors than in the control tumors. It was evident that α-N-catenin significantly reduced tumor cell proliferation and thus inhibited tumor growth and angiogenesis in SK-N-AS xenograft tumors. Same experiments performed using BE(2)-C xenograft tumors, tumor suppression of α-N-catenin was not significant (Figure 4E, tumor volume, left; tumor mass, right), even though increased α-N-catenin was detected in tumor tissue with overexpressed CTNNA2, and no difference on NSE expression between control and CTNNA2 tumor tissues (Figure 4F). However, the number of mitosis cell in CTNNA2 tumor tissue was decreased significantly, approximately 73% of control tumors (Figure 4G). Together, these results demonstrate that restoration of CTNNA2 expression is an advantageous strategy for *in vivo* neuroblastoma growth inhibition and tumor angiogenesis suppression in non-MYCN-amplified tumors, but less effective in MYCN-amplified tumors. Our data was consistent with the analyzed data from patient gene profiling datasets in the R2 platform (Figure 1H, and 1J).

### NF-κB signaling as the key mechanism involved in the tumor suppressor role of α-N-catenin in neuroblastoma

**Figure 5:**
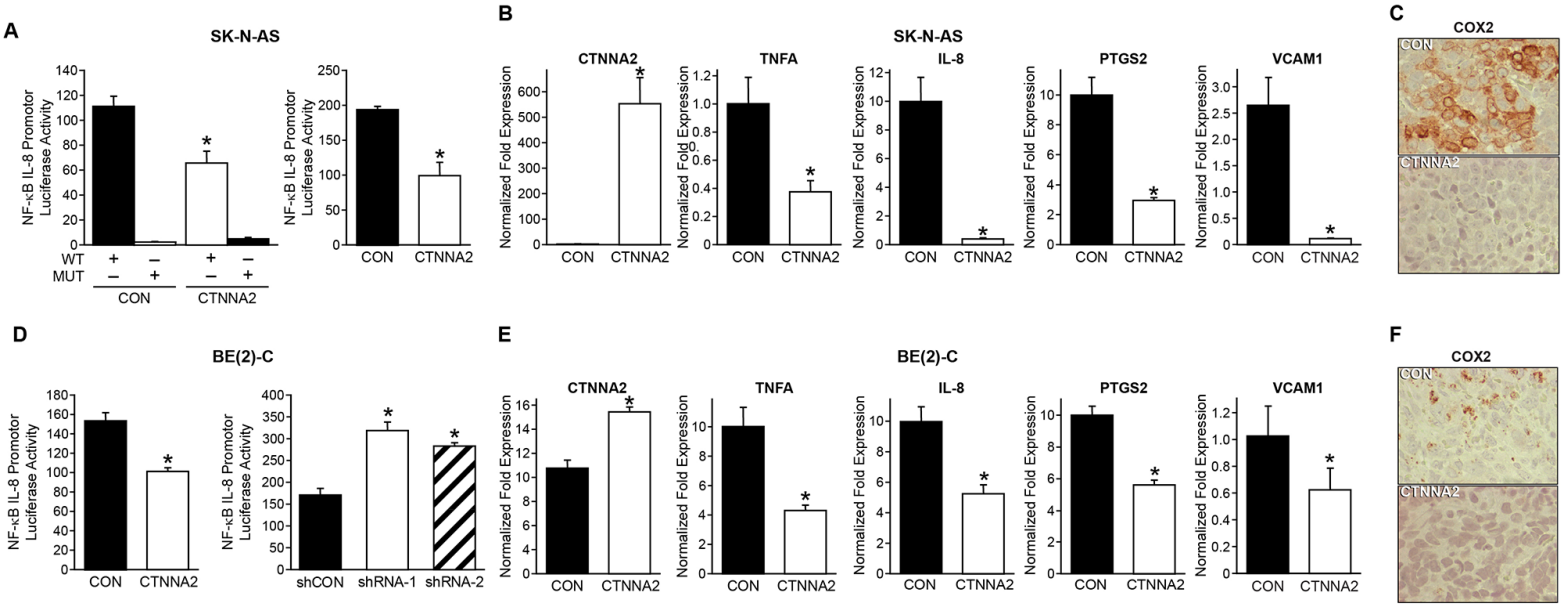
α-N-catenin inhibits NF-κB signaling in neuroblastoma cells. **(A)** Luciferase assays of NF-κB activity were carried out in SK-N-AS cells transfected with plasmid NF-κB luciferase report with wild type (WT) binding site or mutant site (MUT) on the promoter of IL8. **(B)** The expression of CTNNA2 and NF-κB responsive genes were measured with qPCR in control and CTNNA2 overexpressed SK-N-AS cells. **(C)** Expression of COX2, the product of PTGS2, was detected by immunohistochemistry in tumor tissues from SK-N-AS xenografts. **(D)** Luciferase assays of NF-κB activity were carried out in BE(2)-C cells transfected with plasmid NF-κB luciferase report with WT binding site or mutant site MUT on the promoter of IL8. **(E)** The expression of CTNNA2 and NF-κB responsive genes were measured with qPCR in control and CTNNA2 overexpressed BE(2)-C cells. N = 3 samples per group. **(F)** Expression of COX2 was detected by immunohistochemistry in tumor tissues from BE(2)-C xenografts. Data represent mean ± SEM; ^*^ = *p* < 0.05 vs. CON.

To better understand the molecular mechanism and potential signaling pathway affected by α-N-catenin in neuroblastoma, we performed R2 KEGG Pathway Analysis using KEGG PathwayFinder by Gene correlation with CTNNA2 (http://r2.amc.nl). Our results indicated that α-N-catenin is functionally linked to the NF-κB pathway (Supplementary Table 1). To confirm this finding, we used an IL-8 luciferase reporter with specific NF-κB binding sites in the promoter region. As anticipated, overexpression of α-N-catenin markedly suppressed the NF-κB luciferase activity in both SK-N-AS and BE(2)-C cells (Figure 5A and 5D). In contrast, knockdown of α-N-catenin increased the NF-κB luciferase activity in BE(2)-C cells (Figure 5D, right graph). Moreover, we performed qPCR analysis of NF-κB target genes: TNFA, IL8, PTGS2, and vascular cell adhesion molecule 1. Our results showed that all NF-κB target gene expressions were downregulated by overexpressed α-N-catenin in cells (Figure 5B and 5E). Furthermore, the reduction of COX2, a product of gene PTGS2, was confirmed with immunohistochemical staining in both xenograft tumor tissue sections (Figure 5C and 5F). Here, we revealed a robust negative correlation between expression of CTNNA2 and the targets of NF-κB signaling. α-N-catenin suppressed tumor growth by inactivating NF-κB signaling pathway.

## DISCUSSION

Cell adhesion molecules play important roles in cancer by regulating intercellular and extracellular matrix interactions in the development of recurrent invasive and distant metastasis in the progression of cancer. Alterations of adhesion properties change the malignancy of tumor cells and allow cells to escape from the primary site and metastasize to secondary sites. In human α-catenin family, CTNNA1 (α-E-catenin) is expressed ubiquitously in normal tissues [22], and it has been shown to regulate tumor initiation and have a suppressive role in tumor progression of intestinal tumorigenesis [23]. Our analyzed results also showed that there was a trend of correlation between CTNNA1 expression and survival in neuroblastoma within the datasets in R2 platform (Figure 1C and 1I), suggesting a potential suppressive role of CTNNA1 in neuroblastoma. The exact mechanism of how CTNNA1 confers survivability in neuroblastoma will be investigated in the future study. CTNNA3 (α-T-catenin) expression is mainly in the heart and testis and is necessary for the formation of stretch-resistant cell-cell adhesion complexes in muscle cells [24]. CTNNA2 expression is neurally restricted [4] and α-N-catenin governs the stability of synaptic contact [5].

In this study, we found that the expression of α-N-catenin was lost in two of eight human neuroblastoma cell lines. By analyzing mRNA expression levels of CTNNAs in Wolf’s dataset in which 498 patient samples were analyzed in R2 platform, we determined that lost or lower expression of α-N-catenin negatively correlates to relapse-free survival probability in human neuroblastomas subgroup of lacking MYNC amplification. We also showed that α-N-catenin inhibits the cell proliferation, anchorage-independent growth, migration/invasion, and the secretion of angiogenic factors IL-8 and HUVEC tubule formation in both non-MYCN-amplified SK-N-AS cells and MYCN amplified BE(2)-C cells *in vitro*. We also demonstrated that α-N-catenin suppressed tumor growth significantly in SK-N-AS cell-xenografted tumors only, not BE(2)-C cell-xenograft tumors by performing animal tumor growth study. Probably BE(2)-C cells have a fully saturated level of endogenous expression of α-N-catenin, therefore, further overexpression of α-N-catenin could not play a tumor suppressive role. However, we demonstrated that α-N-catenin conversely regulated NF-κB transcriptional activity by luciferase activity assay with overexpression and knockdown of CTNNA2 in BE(2)-C cells (Figure 5D). In addition, we are interested in future knockdown or knockout studies of CTNNA2 from BE(2)-C cells *in vivo*.

The striking heterogeneity of neuroblastoma has been demonstrated by many studies [25]. In order to reproduce the same tumor growth study, we chose another two cell lines in addition to SK-N-AS cells: SK-N-SH and SHEP, both of which do not express α-N-catenin. Forced overexpression of α-N-catenin led to remarkable growth inhibition of SK-N-SH cells, even though we could not successfully establish stably transfected cells. However, we could build a stable transfected α-N-catenin expression cell line in SHEP, but it has not been possible to perform animal tumor growth studies with SHEP cells.

The progression of several human cancers correlates with the loss of the cytoplasmic protein α-catenin from E-cadherin-rich intercellular junctions and subsequent loss of adhesion [26]. However, the potential direct role of α-catenin in modulating the adhesive function of individual E-cadherin or N-cadherin molecules in human cancer is unknown. In breast cancer cells, loss of α-catenin alone drastically reduces the adhesive force between individual cadherin pairs on adjoining cells, explains the global decline of cell adhesion in human breast cancer cells, and shows that the forced expression of α-N-catenin in cancer cells can restore both higher intercellular avidity and intercellular E-cadherin bond strength [26].

In addition to mediating cell-cell interactions, α-catenin regulates intracellular signaling transduction. In this study, we found that α-N-catenin inhibits NF-κB activity. Studies have found aberrant activation of NF-κB signaling in various cancers including neuroblastoma [27, 28, 29]. The activation of NF-κB induces the expression of multiple molecules, including cyclooxygenase-2, matrix metallopeptidase-9 and adhesion molecule intracellular adhesion molecule 1, vascular cell adhesion molecule 1 and endothelial-leukocyte adhesion molecule 1, all of which are linked to cancer cell invasion and metastasis [30]. Our results showed that α-N-catenin reduced the expression of NF-κB downstream targets, such as IL-8, PTGS2, vascular cell adhesion molecule 1, and TNF-α, an activator, and target of NF-κB signaling [31]. Although the mechanism of α-N-catenin lost in neuroblastoma was not determined, it could be related to these epigenetic modifications or other silencing mechanisms. Other groups have found that CTNNA2-promoter hypermethylation is associated with human papillomavirus infection in pharyngeal cancer [32]. Rescue the expression of α-N-catenin is a potential therapeutic strategy for treating patients with neuroblastoma. In conclusion, our study indicated that loss of α-N-catenin exists in a subgroup of neuroblastoma and correlates with relapse-free survival in patients lacking MYCN amplification. We believe that our study could improve the understanding of the molecular mechanisms about α-N-catenin in neuroblastoma, and will lead to a potential novel and personalized therapy in neuroblastomas.

## MATERIALS AND METHODS

### Antibodies and reagents

Primary antibodies α-N-catenin, and p-H3 antibo-dies were from Cell Signaling Technology (Danvers, MA). Primary antibodies NSE (Neuron-Specific Enolase) and CD31/PECAM-1 were obtained from Abcam (Cambridge, MA). Secondary goat anti-mouse and anti-rabbit antibodies were obtained from Santa Cruz Biotechnology, Inc. (Santa Cruz, CA). Secondary goat anti-Human antibody tagged with Alexa Fluor 568 was from Life Technologies (Grand Island, NY). Primary β-actin antibody and all other reagents were obtained from Sigma (St. Louis, MO).

### Cells and cell culture

All neuroblastoma cell lines BE(2)-C, SK-N-AS, BE-M17, SK-N-SH, and SH-SY5Y were purchased from the American Type Culture Collection (ATCC, Manassas, VA). Cells were maintained in RPMI 1640 with 10% Fetal Bovine Serum (FBS) at 37°C in a humidified atmosphere consisting of 5% CO_2_ and 95% air. HUVECs obtained from Dr. M Freeman (Vanderbilt University Medical Center, Nashville, TN) were cultured in EMM-2 supplemented with growth factors (EGM-2 Single- Quot kit, Lonza, Walkersville, MD, USA) at 37°C and humidified 5% CO_2_.

### Plasmids, siRNA and transfections

Plasmid pCMV3-CON (untagged) and pCMV3-CTNNA2 (human NM_004389.3) were ordered from Sino Biological Inc (Beijing, China). Plasmid pLKO.1-shCTNNA2 and its control vector SHC002 (shCON) were purchased from Sigma. siRNA pools targeting CTNNA2 (siCTNNA2) and nontargeting control siRNA (siNTC) were from Dharmacon, Inc (Lafayette, CO). Human neuroblastoma cells were transfected with plasmids or siRNA using Lipofectamine 2000 (Life Technologies) according to the manufacturer’s instructions. Stably-transfected SK-N-AS/CTNNA2 and BE(2)-C/CTNNA2 cells were selected with hygromycin (Sigma) at 300 μg/ml for two weeks. IL-8 promoter luciferase plasmid with specific NF-κB binding sites was kindly provided by Dr. Krach Michael (Institute of Pharmacology, Medical School Hannover, Hanover, Germany).

### Clonogenesis assay

Cells were plated at clonal density (1,000–5,000 cells/well of 6-well plate) in triplicate, permitted to attach and grow for 7–10 days period. Colonies were stained with 0.05% crystal violet, photographed, and counted.

### Soft agar colony formation assay

Cells were trypsinized and resuspended in RPMI medium 1640 containing 0.4% agarose and 5% FBS. BE(2)-C cells were overlaid onto a bottom layer of solidified 0.8% agarose in RPMI medium 1640 containing 5% FBS and incubated for 2 weeks. Colonies were stained with 0.05% crystal violet, photographed, and quantified.

### Migration and invasion assays

For transwell migration, transwell filters (8 μm; Corning, Lowell, MA) were coated on the lower chamber with 5 μg/ml collagen type I (BD Biosciences) overnight and then blocked with 2.5% BSA/PBS for 1 h. 1×10^5^ cells in serum-free media were added to the upper chamber and 10% FBS containing RPMI media into the bottom well and incubated for 6 h. Cells were fixed with 4% paraformaldehyde, stained with DAPI, and counted. For invasion assay, the transwell filters were coated with 1/30 diluted Matrigel (BD Biosciences). Cells (1.5×10^5^) in serum-free media were added to the upper well and 10% FBS containing RPMI media was added to the bottom well. After 48 h incubation, cells were fixed with 4% paraformaldehyde, stained with DAPI, and counted. The assay was performed in duplicate, and cells were counted from five randomly selected microscopic fields.

### Endothelial cell tubule formation assay

HUVECs grown to ~70% confluence were trypsinized, counted, and seeded with 48,000 cells per well in 24-well plates coated with 300 μl of Matrigel (BD Biosciences). These cells were periodically observed by a microscope as they differentiated into capillary-like tubule structures. After 6 h, the cells were stained with hematoxylin and eosin and photographs were taken by a microscope, and representative pictures obtained. Tubules were developed from clear elongated cell bodies that connect to form a polygon network. The average number of tubules was quantified by randomly selecting three separate ×200 fields and counting the number of tubules per field.

### RNA isolation and qPCR with reverse transcription

Total RNA was isolated and purified using a RNeasy isolation kit (Qiagen, Germantown, MD) with DNase digestion. cDNA was synthesized using the High-Capacity cDNA Reverse Transcription Kit (Applied Biosystems, Carlsbad, CA). Real-time PCR and data collection were performed on a CFX96 instrument (Bio-Rad). Data were normalized to an endogenous control, GAPDH. Specific target primers are listed in Supplementary Table 2. Amplification was performed for 40 cycles of 30 s at 95°C, 30 s at 55°C, and 40 s at 72°C.

### Immunoblotting

Cells were collected using cell lysis buffer, and denatured samples were prepared for immunoblotting. Equal amounts of protein were loaded and separated by NuPAGE 4–12% Bis-Tris gel, followed by transfer onto PVDF membranes (Bio-Rad, Hercules, CA, USA). Membranes were blocked with 5% nonfat milk in TBS-T for 1 h at room temperature. The blots were then incubated with antibodies against the human target proteins by using rabbit or mouse anti-human antibodies (1:500– 2000 dilution) overnight at 4°C. Anti-rabbit or anti-mouse secondary antibodies conjugated with HRP was incubated for 1 h and visualized using an enhanced chemiluminescence detection system (PerkinElmer, Waltham, MA, USA).

### Enzyme-linked immunosorbent assay (ELISA)

Cells were plated at (3×10^5^ cells/ well) were plated in 6-well plate and cultured in 10% FBS-containing medium for 24 h, washed with PBS and then incubated with serum-free medium. After a 24-h or 48-h incubation, conditioned medium was collected for ELISA using the DuoSet ELISA Development System (IL8: DY208, R&D Systems, Minneapolis, MN) according to the manufacturer's protocol.

### Luciferase reporter assay

Cells of 70% confluence in 24-well plates were transfected using Lipofectamine 2000 (LifeTechnology). The firefly luciferase reporter gene construct (0.2 μg) and the pRL-SV40 *Renilla* luciferase construct (20 ng, for normalization) were used for co-transfection. Cell extracts were prepared 24–48 h after transfection and the luciferase activity was measured using the Dual-Luciferase Reporter Assay System (Promega, Madison, WI).

### Immunohistochemistry and immunofluorescence staining

Immunohistochemical staining was performed using DAKO EnVision+ System-HRP from Dako North America, Inc. (Carpinteria, CA). Mouse neuroblastoma xenografts were fixed in formalin overnight and embedded in paraffin wax. Tumor sections (5 mm) were mounted on glass slides. Samples were deparaffinized and rehydrated. The antigen was retrieved using 0.01 M sodium-citrate buffer (pH 6.0) at a sub-boiling temperature for 10 min after boiling in a microwave oven. To block endogenous peroxidase activity, the sections were incubated with 3% hydrogen peroxide for 5 min. After 1 h of pre-incubation in 5% normal goat serum to prevent nonspecific staining, the samples were incubated with the antibody against α-N-catenin, NSE, or CD31 at 4°C overnight. They were then washed with buffer three times for 5 min each and incubated with secondary antibody for 30 min at room temperature. Sections were developed with the DAB reagent. The reaction was terminated by immersing slides in dH_2_O and sections were counterstained with hematoxylin. Slides were then dehydrated with ethanol and xylene. Coverslips were mounted and slides were left to dry. The IHC images were taken under a microscope (Leica DMI6000 B). For mitosis detection, paraffin-embedded sections were stained with anti-human phospho-Histone H3 (Ser10) antibody followed by Alexa Fluor 568 Dye (Life Technologies, Grand Island, NY). DAPI was used for staining nuclei. Images were captured using a fluorescence microscope (Nikon Eclipse E600).

### Murine xenograft models

Male athymic nude mice (4–6 weeks old) were maintained as described [33]. All studies were approved by the Institutional Animal Care and Use Committee at Vanderbilt University. SK-N-AS and BE(2)-C cells xenografts were established as previously described [33]. Briefly, 1 × 10^6^ cells/100 μL of HBSS were injected subcutaneously into flanks using a 26-gauge needle (*n* = 10 per group). Mice were monitored daily for xenograft formation and assessed by measuring the two greatest perpendicular tumor diameter with vernier calipers (Mitutoyo, Aurora, IL). Xenograft volumes were estimated using the following formula [(length × width^2^)/2]. Weight and tumor volume were recorded biweekly. At four weeks of post-injection, mice were euthanized when they met the institutional euthanasia criteria for tumor size and overall health condition. The tumors were excised, weighed, and fixed in 10% buffered formalin. Tumor tissues were further processed for embedding in paraffin, sectioned and stained with hematoxylin and eosin at Translational Pathology Shared Resource Laboratory in Vanderbilt University Medical Center.

### Statistical analysis and gene expression and signaling pathway analysis

The scoring index was expressed as means ± SEM; statistical analyses were performed using student t-test for comparisons between the groups. A *p* value of < 0.05 was considered significant. R2 is a genomics analysis and visualization platform developed in the Department of Oncogenomics at the Academic Medical Center University of Amsterdam (http://r2.amc.nl). We performed the analysis correlation of the expression levels of CTNNAs to relapse-free survival probability using the Kaplan-Meier scan (cutoff value was decided on an equally split number of patients into two groups from two different datasets: Wolf’s dataset (498 samples) and Seeger’s dataset (102 samples lacking MYCN amplification). For mRNA gene expression levels and associated potential pathways were analyzed using bioinformatic platform R2 which connected with the KEGG pathway database (http://www.genome.jp/kegg/pathway.html).

## SUPPLEMENTARY MATERIALS FIGURES AND TABLES





## Abbreviations

CTNNA1: Catenin Alpha 1
CTNNA2: Catenin Alpha 2
CTNNA3: Catenin Alpha 3
MYCN: Neuroblastoma MYC Oncogene
NF-κB: Nuclear Factor Kappa B
CD31/PECAM-1: Platelet Endothelial Cell Adhesion Molecule-1
HUVEC: Human umbilical vein endothelial cells
FBS: Fetal Bovine Serum
CON: Control
ELISA: Enzyme-Linked Immunosorbent Assay
siNTC: small interfering RNA nontargeting control
shCON: small hairpin RNA nontargeting control
siCTNNA2: siRNA pools targeting CTNNA2
WT: wild type
MUT: mutant site.

## References

[1] Matthay KK , Maris JM , Schleiermacher G , Nakagawara A , Mackall CL , Diller L , Weiss WA . Neuroblastoma. Nat Rev Dis Primers. 2016; 2:16078. 10.1038/nrdp.2016.78. 27830764

[2] Durinck K , Speleman F . Epigenetic regulation of neuroblastoma development. Cell Tissue Res. 2018; 372:309–324. 10.1007/s00441-017-2773-y. 29350283

[3] Maiden SL , Hardin J . The secret life of alpha-catenin: moonlighting in morphogenesis. J Cell Biol. 2011; 195:543–552. 10.1083/jcb.201103106. 22084304 PMC3257527

[4] Uchida N , Shimamura K , Miyatani S , Copeland NG , Gilbert DJ , Jenkins NA , Takeichi M . Mouse alpha N-catenin: two isoforms, specific expression in the nervous system, and chromosomal localization of the gene. Dev Biol. 1994; 163:75–85. 10.1006/dbio.1994.1124. 8174789

[5] Abe K , Chisaka O , Van Roy F , Takeichi M . Stability of dendritic spines and synaptic contacts is controlled by alpha N-catenin. Nat Neurosci. 2004; 7:357–363. 10.1038/nn1212. 15034585

[6] Inge LJ , Rajasekaran SA , Wolle D , Barwe SP , Ryazantsev S , Ewing CM , Isaacs WB , Rajasekaran AK . alpha-Catenin overrides Src-dependent activation of beta-catenin oncogenic signaling. Mol Cancer Ther. 2008; 7:1386–1397. 10.1158/1535-7163.MCT-07-2029. 18566211 PMC2527861

[7] Hollestelle A , Elstrodt F , Timmermans M , Sieuwerts AM , Klijn JG , Foekens JA , den Bakker MA , Schutte M . Four human breast cancer cell lines with biallelic inactivating alpha-catenin gene mutations. Breast Cancer Res Treat. 2010; 122:125–133. 10.1007/s10549-009-0545-4. 19763817

[8] Fanjul-Fernandez M , Quesada V , Cabanillas R , Cadinanos J , Fontanil T , Obaya A , Ramsay AJ , Llorente JL , Astudillo A , Cal S , Lopez-Otin C . Cell-cell adhesion genes CTNNA2 and CTNNA3 are tumour suppressors frequently mutated in laryngeal carcinomas. Nat Commun. 2013; 4:2531. 10.1038/ncomms3531. 24100690

[9] Kobielak A , Fuchs E . α-catenin: at the junction of intercellular adhesion and actin dynamics. Nat Rev Mol Cell Biol. 2004; 5:614–625. 10.1038/nrm1433. 15366705 PMC2475680

[10] Vasioukhin V , Bauer C , Degenstein L , Wise B , Fuchs E . Hyperproliferation and defects in epithelial polarity upon conditional ablation of alpha-catenin in skin. Cell. 2001; 104:605–617. 10.1016/S0092-8674(01)00246-X. 11239416

[11] Yue D , Li H , Che J , Zhang Y , Tseng HH , Jin JQ , Luh TM , Giroux-Leprieur E , Mo M , Zheng Q , Shi H , Zhang H , Hao X , et al. Hedgehog/Gli promotes epithelial-mesenchymal transition in lung squamous cell carcinomas. J Exp Clin Cancer Res. 2014; 33:34. 10.1186/1756-9966-33-34. 24758269 PMC4029998

[12] Kim NG , Koh E , Chen X , Gumbiner BM . E-cadherin mediates contact inhibition of proliferation through Hippo signaling-pathway components. Proc Natl Acad Sci U S A. 2011; 108:11930–11935. 10.1073/pnas.1103345108. 21730131 PMC3141988

[13] Schlegelmilch K , Mohseni M , Kirak O , Pruszak J , Rodriguez JR , Zhou D , Kreger BT , Vasioukhin V , Avruch J , Brummelkamp TR , Camargo FD . Yap1 acts downstream of alpha-catenin to control epidermal proliferation. Cell. 2011; 144:782–795. 10.1016/j.cell.2011.02.031. 21376238 PMC3237196

[14] Sehgal RN , Gumbiner BM , Reichardt LF . Antagonism of cell adhesion by an alpha-catenin mutant, and of the Wnt-signaling pathway by alpha-catenin in Xenopus embryos. J Cell Biol. 1997; 139:1033–1046. 10.1083/jcb.139.4.1033. 9362521 PMC2139960

[15] Gottardi CJ , Gumbiner BM . Distinct molecular forms of beta-catenin are targeted to adhesive or transcriptional complexes. J Cell Biol. 2004; 167:339–349. 10.1083/jcb.200402153. 15492040 PMC2172558

[16] Piao HL , Yuan Y , Wang M , Sun Y , Liang H , Ma L . alpha-catenin acts as a tumour suppressor in E-cadherin-negative basal-like breast cancer by inhibiting NF-kappaB signalling. Nat Cell Biol. 2014; 16:245–254. 10.1038/ncb2909. 24509793 PMC3943677

[17] Kocak H , Ackermann S , Hero B , Kahlert Y , Oberthuer A , Juraeva D , Roels F , Theissen J , Westermann F , Deubzer H , Ehemann V , Brors B , Odenthal M , et al. Hox-C9 activates the intrinsic pathway of apoptosis and is associated with spontaneous regression in neuroblastoma. Cell Death Dis. 2013; 4:e586. 10.1038/cddis.2013.84. 23579273 PMC3668636

[18] Molenaar JJ , Koster J , Zwijnenburg DA , van Sluis P , Valentijn LJ , van der Ploeg I , Hamdi M , van Nes J , Westerman BA , van Arkel J , Ebus ME , Haneveld F , Lakeman A , et al. Sequencing of neuroblastoma identifies chromothripsis and defects in neuritogenesis genes. Nature. 2012; 483:589–593. 10.1038/nature10910. 22367537

[19] Friedl P , Gilmour D . Collective cell migration in morphogenesis, regeneration and cancer. Nat Rev Mol Cell Biol. 2009; 10:445–457. 10.1038/nrm2720. 19546857

[20] Matsuzawa K , Himoto T , Mochizuki Y , Ikenouchi J . alpha-Catenin Controls the Anisotropy of Force Distribution at Cell-Cell Junctions during Collective Cell Migration. Cell Rep. 2018; 23:3447–3456. 10.1016/j.celrep.2018.05.070. 29924989

[21] Vassilev V , Platek A , Hiver S , Enomoto H , Takeichi M . Catenins Steer Cell Migration via Stabilization of Front-Rear Polarity. Dev Cell. 2017; 43:463–479.e5. 10.1016/j.devcel.2017.10.014. 29103954

[22] Furukawa Y , Nakatsuru S , Nagafuchi A , Tsukita S , Muto T , Nakamura Y , Horii A . Structure, expression and chromosome assignment of the human catenin (cadherin-associated protein) alpha 1 gene (CTNNA1). Cytogenet Cell Genet. 1994; 65:74–78. 10.1159/000133603. 8404069

[23] Shibata H , Takano H , Ito M , Shioya H , Hirota M , Matsumoto H , Kakudo Y , Ishioka C , Akiyama T , Kanegae Y , Saito I , Noda T . Alpha-catenin is essential in intestinal adenoma formation. Proc Natl Acad Sci U S A. 2007; 104:18199–18204. 10.1073/pnas.0705730104. 17989230 PMC2084320

[24] Janssens B , Goossens S , Staes K , Gilbert B , van Hengel J , Colpaert C , Bruyneel E , Mareel M , van Roy F . alphaT-catenin: a novel tissue-specific beta-catenin-binding protein mediating strong cell-cell adhesion. J Cell Sci. 2001; 114:3177–3188. 11590244 10.1242/jcs.114.17.3177

[25] Boeva V , Louis-Brennetot C , Peltier A , Durand S , Pierre-Eugene C , Raynal V , Etchevers HC , Thomas S , Lermine A , Daudigeos-Dubus E , Geoerger B , Orth MF , Grunewald TGP , et al. Heterogeneity of neuroblastoma cell identity defined by transcriptional circuitries. Nat Genet. 2017; 49:1408–1413. 10.1038/ng.3921. 28740262

[26] Bajpai S , Feng Y , Krishnamurthy R , Longmore GD , Wirtz D . Loss of alpha-catenin decreases the strength of single E-cadherin bonds between human cancer cells. J Biol Chem. 2009; 284:18252–18259. 10.1074/jbc.M109.000661. 19458087 PMC2709389

[27] Karin M . Nuclear factor-kappaB in cancer development and progression. Nature. 2006; 441:431–436. 10.1038/nature04870. 16724054

[28] Rayet B , Gelinas C . Aberrant rel/nfkb genes and activity in human cancer. Oncogene. 1999; 18:6938–6947. 10.1038/sj.onc.1203221. 10602468

[29] Zhi Y , Lu H , Duan Y , Sun W , Guan G , Dong Q , Yang C . Involvement of the nuclear factor-kappaB signaling pathway in the regulation of CXC chemokine receptor-4 expression in neuroblastoma cells induced by tumor necrosis factor-alpha. Int J Mol Med. 2015; 35:349–357. 10.3892/ijmm.2014.2032. 25503960 PMC4292717

[30] Sethi G , Tergaonkar V . Potential pharmacological control of the NF-kappaB pathway. Trends Pharmacol Sci. 2009; 30:313–321. 10.1016/j.tips.2009.03.004. 19446347

[31] Schutze S , Wiegmann K , Machleidt T , Kronke M . TNF-induced activation of NF-kappa B. Immunobiology. 1995; 193:193–203. 10.1016/S0171-2985(11)80543-7. 8530143

[32] Nakagawa T , Matsusaka K , Misawa K , Ota S , Takane K , Fukuyo M , Rahmutulla B , Shinohara KI , Kunii N , Sakurai D , Hanazawa T , Matsubara H , Nakatani Y , et al. Frequent promoter hypermethylation associated with human papillomavirus infection in pharyngeal cancer. Cancer Lett. 2017; 407:21–31. 10.1016/j.canlet.2017.08.008. 28823962

[33] Kang J , Ishola TA , Baregamian N , Mourot JM , Rychahou PG , Evers BM , Chung DH . Bombesin induces angiogenesis and neuroblastoma growth. Cancer Lett. 2007; 253:273–281. 10.1016/j.canlet.2007.02.007. 17383815 PMC2709810

