# Supplementary material for: Identification of α-N-catenin as a novel tumor suppressor in neuroblastoma
Source: Oncotarget. 2019 Aug 20;10(49):5028–40. doi: 10.18632/oncotarget.27096 (PMC6707940; doi:10.18632/oncotarget.27096)
Supplement: Supplementary file 1 [file oncotarget-10-5028-s001.pdf]

Identification of α-N-catenin as a novel tumor suppressor in neuroblastoma

SUPPMENTARY MATERIALS

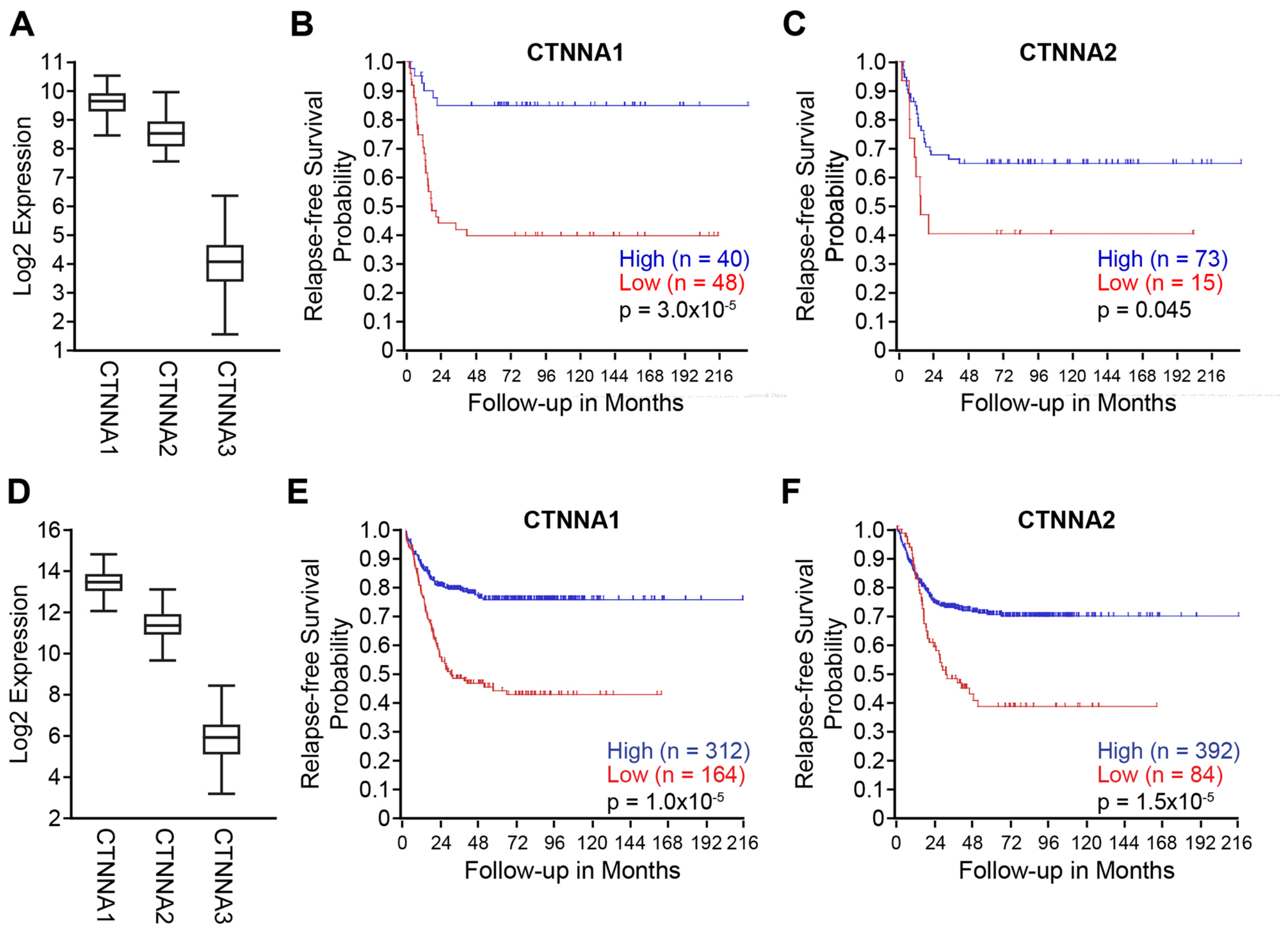

Supplementary Figure 1: Low α-catenin expression is associated with disease relapse and mortality in patients with neuroblastoma. Analyzed using the publicly available microarray gene expression Versteeg and Kocak datasets downloaded from the R2 analysis and visualization platform (http://r2.amc.nl). **(A, B** and **C)** Versteeg dataset, 88 human neuroblastoma samples. (A) The expression levels of three CTNNA family members were analyzed. (B, C) Kaplan–Meier curves showed the probability of relapse-free survival are correlated to levels of CTNNA1 and CTNNA2 mRNA expression. **(D, E** and **F)** Kocak dataset, 649 human neuroblstoma samples. (D) The expression levels of three CTNNA family members were analyzed. (E, F) Kaplan–Meier curves showed the probability of relapse-free survival are correlated to levels of CTNNA1 and CTNNA2 mRNA expression.

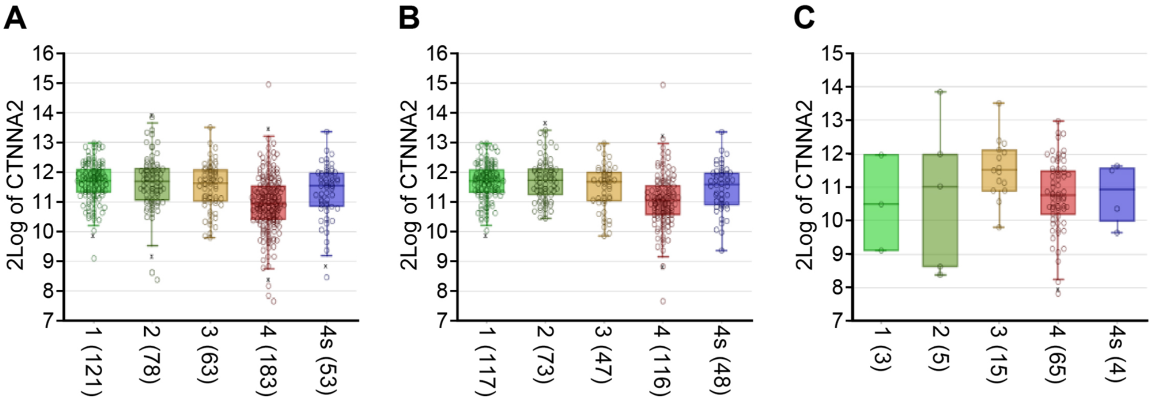

A

Combin-ation	T Prob-ability	Remarks
1 vs 2	0.91	var not equal tutest=-0.91
1 vs 3	0.18	–
1 vs 4	**5.0e-12**	var not equal tutest=**1.3e-13**
1 vs 4s	0.01	var not equal tutest=0.03
2 vs 3	0.35	–
2 vs 4	**7.1e-08**	–
2 vs 4S	0.07	–
3 vs 4	**3.0e-05**	–
3 vs 4s	0.32	–
4 vs 4s	**5.3e-09**	–

B

Combin-ation	T Prob-ability	Remarks
1 vs 2	0.81	–
1 vs 3	0.08	–
1 vs 4	**1.6e-09**	var not equal tutest=**2.1e-09**
1 vs 4s	0.06	–
2 vs 3	0.10	–
2 vs 4	**3.5e-07**	–
2 vs 4S	0.08	–
3 vs 4	**4.2e03**	–
3 vs 4s	0.89	–
4 vs 4s	**6.3e-03**	–

C

Combin-ation	T Prob-ability	Remarks
1 vs 2	0.88	–
1 vs 3	0.11	–
1 vs 4	0.71	–
1 vs 4s	0.78	var not equal
2 vs 3	0.28	tutest=0.50
		var not equal
2 vs 4	0.99	tutest=0.99
		–
2 vs 4S	0.99	–
3 vs 4	**9.0e03**	–
3 vs 4s	0.15	–
4 vs 4s	0.97	–

Supplementary Figure 2: Low α-N-catenin expression is associated with clinical high-risk neuroblastoma. We analyzed the CTNNA2 expression and stratified the survival data based on the clinical risk groups of patients in Wolf's dataset. **(A)** In overall 498 samples, the expression of CTNNA2 in stage 4 was significantly lower compared to other groups, stage 1, stage 2, stage 3 and stage 4S. **(B)** In non-MYCN amplified 401 samples, the expression of CTNNA2 in stage 4 was significantly lower compared to other groups, stage 1, stage 2, stage 3 and stage 4S. **(C)** In MYCN amplified 92 samples, the expression of CTNNA2 in stage 4 was lower than the group of stage 3. The lower number of sample in each group is the limitation to make conclusion. The *p* values are listed in the bottom tables.

Supplementary Table 1: NF-κB pathway is functionally linked to α-N-catenin in R2 KEGG Pathway. Analyzed using KEGG PathwayFinder by Gene correlation with CTNNA2 (http://r2.amc.nl).

See Supplementary File 1

Supplementary Table 2: Primers and their sequences were used in qRT-PCR

Gene	Primer (5' - 3')
CTNNA2	Forward: AATTAGCTCGTGCTGTGGCT
	Reverse: ACCGTGAGGACAACAGCATT
TNF-α	Forward: CCCAGGCAGTCAGATCATCTTC
	Reverse: AGCTGCCCCTCAGCTTGA
IL-8	Forward: TTTTGCCAAGGAGTGCTAAAGA
	Reverse: AACCCTCTGCACCCAGTTTTC
PTGS2	Forward: GGCTTCCATTGACCAGAGCAG
	Reverse: GCCGAGGCTTTTCTACCAGA
VCAM1	Forward: TGGCATGGTACGGAGATGTT
	Reverse: GCTACAACGTGACTAAAGGAGG
