# Supplementary material for: Identification of α-N-catenin as a novel tumor suppressor in neuroblastoma
Source: Oncotarget. 2019 Aug 20;10(49):5028–40. doi: 10.18632/oncotarget.27096 (PMC6707940; doi:10.18632/oncotarget.27096)
Supplement: Supplementary file 2 [file oncotarget-10-5028-s002.docx]

**Supplemental Table 1. NF-κB pathway is functionally linked to α-N-catenin in R2 KEGG Pathway. Analyzed using KEGG PathwayFinder by Gene correlation with CTNNA2 (http://r2.amc.nl).**

| **GROUP** | **IN_SET** | **TOTAL** | **PERCENTAGE** | **P-VALUE** |
| --- | --- | --- | --- | --- |
| **All** | **1882** | **4356** | **43.2%** | **–** |
| NF_Kappa_B_signaling _ pathway | 55 | 73 | 75.3% | 3.0e-08 |
| Hematopoietic_cell_lineage | 47 | 62 | 75.8% | 2.2e-07 |
| Rheumatoid_arthritis | 47 | 63 | 74.6% | 4.9e-07 |
| Cytokine_cytokine_receptor_interaction | 99 | 157 | 63.1% | 5.1e-07 |
| Intestinal_immune_network_for_IgA_production | 26 | 30 | 86.7% | 1.5e-06 |
| TNF_signaling_pathway | 63 | 93 | 67.7% | 1.8e-0.6 |
